# Effective treatment of bronchopleural fistula with empyema by pedicled latissimus dorsi muscle flap transfer: Two case report

**DOI:** 10.1097/MD.0000000000022485

**Published:** 2020-10-09

**Authors:** Zhongliang He, Lifeng Shen, Weihua Xu, Xiaowen He

**Affiliations:** a Department of Cardiothoracic Surgery; b Department of Traumatology and Orthopedic Surgery; c Department of Interventional Pulmonology, Tongde Hospital of Zhejiang Province; d Division of Endocrinology and Metabolism, Department of Medicine, 2nd Affiliated Hospital of Zhejiang University Medical School, Hangzhou, Zhejiang, China.

**Keywords:** bronchopleural fistula, empyema, muscle flap, latissimus dorsi muscle

## Abstract

**Rationale:**

Bronchopleural fistula (BPF) is a dreaded complication after lobectomy or pneumonectomy and is associated with high morbidity and mortality. Successful management remains challenging when this condition is combined with empyema, and the initial treatment is usually conservative and endoscopic, but operative intervention may be required in refractory cases.

**Patient concerns:**

Two patients diagnosed with BPF with empyema were selected to undergo surgery in our hospital because they could not be cured by conservative and endoscopic therapy for 1 or more years. One was a 70-year-old man who had a 1-year history of fever and cough after he received a minimally invasive right lower lobectomy for intermediate lung adenocarcinoma and chemotherapy 2 years ago; the other was a 73-year-old man who had a 2-year history of cough and fever after he underwent a minimally invasive right upper lobectomy for early lung adenocarcinoma 3 years earlier.

**Diagnosis:**

Both patients were diagnosed with BPF with empyema.

**Interventions:**

After receiving conservative and endoscopic therapies, both patients underwent pedicled latissimus dorsi muscle flap transfers for complete filling of the empyema cavity.

**Outcomes:**

The patients recovered very well, with no recurrence of BPF and empyema during postoperative follow-up.

**Lessons:**

It is crucial to not only completely control infection and occlude BPFs, but also obliterate the empyema cavity. Thus, pedicled latissimus dorsi muscle flap transfer associated with conservative and endoscopic therapies for BPF with empyema is a useful treatment option, offering feasible and efficient management with promising results.

## 1 Introduction

Bronchopleural fistula (BPF) is an uncommon, but severe complication of lobectomy or pneumonectomy and is associated with high morbidity and mortality rates.^[1,2]^ Although there are a wide variety of treatment options for BPFs, successful management remains challenging when this condition is combined with refractory empyema because, not only is the infection difficult to control, but the residual space and fistula easily redevelop. Therefore, operative intervention, combined with conservative and endoscopic therapies, may be required to completely control infection, occlude bronchopleural fistulae, and obliterate the empyema cavity in refractory cases during treatment periods.^[3,4]^ In this study, we describe the successful treatment of 2 patients diagnosed with BPF with empyema using thoracostomy and endobronchial stenting, and followed by pedicled latissimus dorsi muscle flap transfer.

## 2 Case presentation

This report of 2 cases was approved by the Institutional Review Board of the Tongde Hospital of Zhejiang Province. Written consent was obtained from both patients for the publication of their cases.

### 2.1 Patient 1

**Figure 1:**
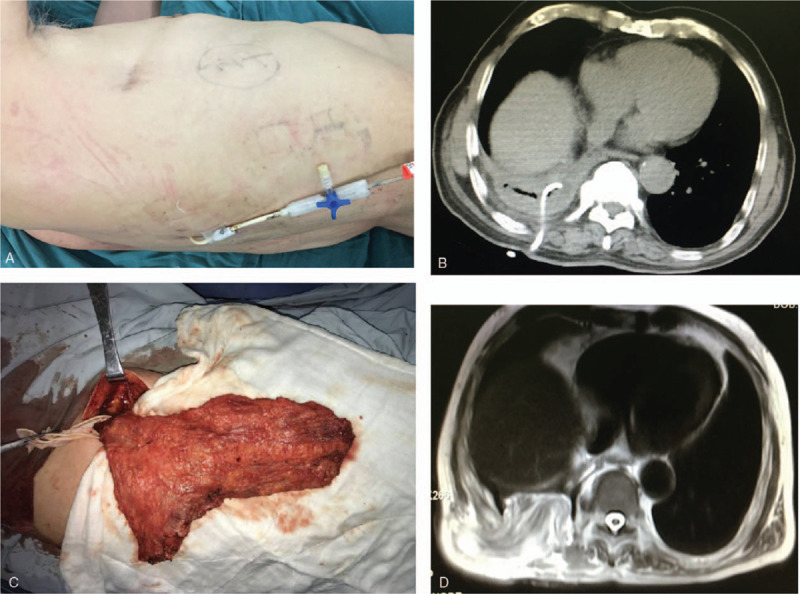
Clinical data of patient 1. A, A preoperative image revealed a uniportal incision and a drainage tube in the right lower thorax. B, A preoperative chest computed tomography (CT) scan showed a empyema cavity containing pleural effusion in the right lower thorax. C, During the operation, a 15 × 11 cm pedicled latissimus dorsi muscle flap was harvested and the tip of the pedicled muscle flap was sutured and fixed to the anterior lower mediastinum and the rest was transposed to completely obliterate the empyema cavity. D, A postoperative chest magnetic resonance imaging (MRI) scan showed successful obliteration of the empyema cavity and bronchopleural fistula BPF after surgery.

**Figure 2:**
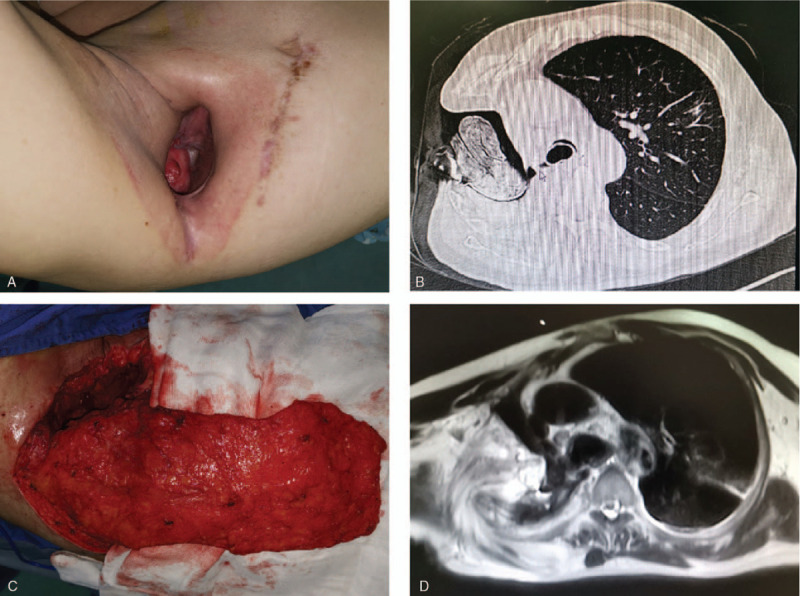
Postoperative images of both patients. A, Postoperative image of ptient 1. B, Postoperative image of patient 2.

The patient was a 70-year-old man with a history of a solitary pulmonary nodule in the right lower lobe. He underwent a minimally invasive right lower lobectomy for lung adenocarcinoma (pT2aN1M0) and chemotherapy 2 years ago. He exhibited no specific symptoms until he was admitted to hospital due to progression of a severe cough with fever, 12 months after lung resection. Although his fever was relieved by antibiotic administration and insertion of closed thoracic drainage, his cough continued and a large volume of air leaked into the chest tube. A physical examination revealed a uniportal incision and a chest tube in the right thorax (Fig. 1A). Chest computed tomography (CT) scan showed a simple empyema cavity containing a pleural effusion and a tube in the right lower thorax (Fig. 1B). Based on his clinical symptoms and these imaging findings, we diagnosed the patient with right lower bronchial stump fistula with empyema after lobectomy. Bronchoscopy confirmed a fistula of approximately 5 mm in diameter between the right lower bronchial stump and pleural cavity, and endoscopic placement of covered bronchial stent (Boston Scientific Corporation, Natick, MA) was performed. After that, air leakage was lessened but discharge of purulent fluid from the tube continued at about 60 mL/day. Due to the inadequate outcome, rib resection thoracostomy was recommended. After the sixth and seventh posterior ribs were partially resected, purulent pleural effusion was observed in the thoracic cavity, which was cleaned to remove debris and necrotic tissue by debridement and washing. Two drainage tubes were inserted into the cavity and the wound was closed. The pathogen identified via pus culturing was *Klebsiella pneumoniae*. After irrigation of the right intrathoracic space with antibiotic solution for 14 days, his condition improved. When cultures confirmed there was no infection in the thoracic cavity, he underwent surgical intervention. During operation, a 15 cm × 11 cm latissimus dorsi muscle flap was harvested, the tip of the pedicled muscle flap was sutured and fixed to the anterior lower mediastinum, and the rest was transposed to completely obliterate the empyema cavity (Fig. 1C). His postoperative course was uneventful, and he was discharged on postoperative day (POD) 21(Fig. 2A). The chest magnetic resonance imaging (MRI) revealed successful obliteration of the empyema cavity and BPF after surgery (Fig. 1D). The patient remained healthy, and no recurrence of the fistula with empyema was evident during the 22 months of follow-up.

### 2.2 Patient 2

**Figure 3:**
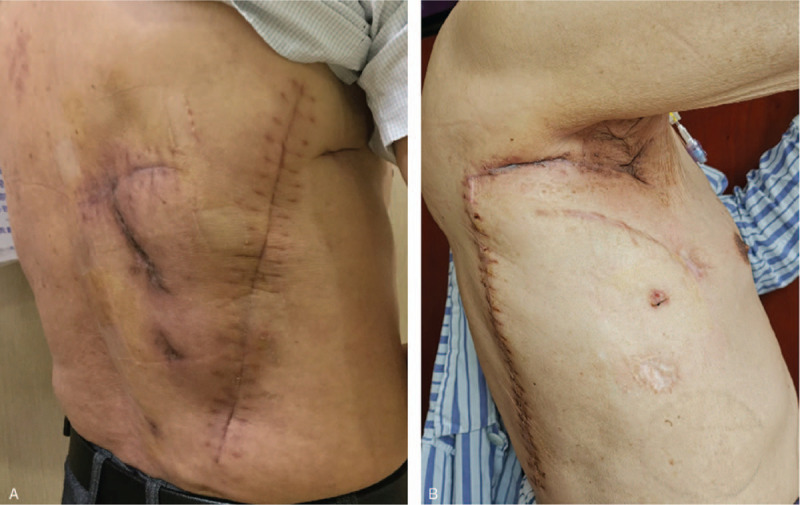
Clinical data of patient 2. A, A preoperative image showed a 6 mm bronchopleural fistula (BPF) which was existed with small leakage and a 10.0 × 8.0 × 5.0 cm thoracic defect in the right upper thorax after an open-window thoracic procedure. B, A preoperative chest computed tomography (CT) scan revealed a huge thoracic defect in the right upper thorax, and a silicone stent in the tracheobronchus. C, During the operation, a 7 × 3 cm surrounding pedicle skin was harvested to repair BPF, and a 20 × 10 cm pedicled latissimus dorsi muscle flap was harvested and transposed to completely obliterate the thoracic defect. D, A postoperative chest magnetic resonance imaging (MRI) scan showed successful repair of BPF by pedicled skin flap and complete obliteration of the thoracic cavity by pedicled muscle flap, and the defect of chest wall been closed as well after surgery.

The patient was a 73-year-old man with a history of ground-glass opacity in the right upper lobe. He underwent a minimally invasive right upper lobectomy for lung adenocarcinoma (pT1aN0M0) 3 years earlier. He had not exhibited any specific symptoms until he was admitted to the hospital due to the progression of severe cough with fever 12 months after lung resection. Although his fever was relieved by the administration of antibiotics and insertion of closed thoracic drainage, his cough continued and there was a large amount of air leakage in the tube. Chest CT scan showed an empyema cavity containing pleural effusion in the right upper thorax. When right lower bronchial fistula with empyema was diagnosed, he underwent open window thoracostomy (OWT) where the incision was made along the previous small incision in the most dependent portion of the empyema cavity and a 6 mm BPF was found in the cavity, and dressing changes and compressive bandage application were done regularly during the subsequent weeks. Then, endoscopic placement of a covered bronchial stent (Boston Scientific Corporation, Natick, MA) was performed but failed, and he underwent re-endoscopic therapy, where a copper silicone stent (Tracheobronxane, Novatech, La Ciotat, France) was placed with the proximal end in the lower trachea and the distal end in the main bronchus contralateral to the fistula, and air leakage was minimized through the BPF. Physical examination revealed a thoracic defect about 10.0 × 8.0 × 5.0 cm in the right thorax (Fig. 3A) . The chest CT scan showed a huge thoracic defect in the right upper thorax, and a silicone stent in the tracheobronchus (Fig. 3B). After a combination of systemic antibiotic therapy and daily dressing applications over several months, the local infection could not be controlled effectively. The pathogen *Pseudomonas aeruginosa* was identified by culture, so vacuum-assisted closure (VAC) therapy (Smith & Nephew Inc, Michigan) was used, and his general condition improved. When healthy granulation tissue appeared in the cavity after 10-day VAC therapy, pedicled muscle transposition was considered. During operation a piece of 7 × 3 cm pedicle skin surrounding the defect was harvested to repair BPF, and a 20 × 10 cm pedicled latissimus dorsi muscle flap was harvested and transposed to completely obliterate the thoracic defect (Fig. 3C). His postoperative course was uneventful. Chest MRI revealed successful repair of BPF and complete obliteration of the thoracic defect after surgery (Fig. 3D). Bronchoscopy on POD 18 revealed successful repair of BPF and subsequently the stent was removed. He was discharged on POD 22 (Fig. 2B). The patient recovered well, and no recurrence of the fistula with empyema was evident during the 8 months of follow-up.

## 3 Discussion

BPF is a communication between the pleural space and the bronchial tree. It is a potentially fatal postoperative complication after pulmonary resection and a complex challenge for thoracic surgeons because many patients with fistulas ultimately have refractory empyema, which is difficult to manage and has a major impact on quality of life and survival.^[1,2]^ The prevalence of BPF after pulmonary resection ranges from 1.5% to 28% and this variability depends on etiology, surgical technique, and experience of the surgeon.^[3,4]^ The etiology of a BPF with empyema is still not completely understood. Local factors may include the technique of stump closure, a long bronchial stump, residual carcinoma at the bronchial margin, disruption of bronchial blood supply, extended resection, right-side resection, pneumonectomy, presence of empyema, and high-dose preoperative radiation therapy; however, no single factor has been clearly identified. Systemic factors included the patient's nutritional status, diabetes mellitus, steroid use, presence of sepsis, and preoperative chemotherapy.^[5,6]^ Patients with BPF typically present 1 to 2 weeks after lung resection with fever, productive cough, purulent or hemorrhagic sputum, respiratory distress, and occasionally sepsis and acute respiratory failure. Patients presenting later, typically present subacutely with malaise, flulike symptoms, low-grade fever, and weight loss. It can lead to persistent contamination and infection of the pleural space, trapped lung, aspiration in the unaffected lung, and even death. The mortality rate associated with BPF after pneumonectomy was reported to range from 20% to 70%. Diagnosis of a BPF is usually confirmed by contrast-enhanced CT scan of the chest or by bronchoscopy.^[7,8]^

Once BPF with empyema has developed, proper and prompt management is mandatory to reduce the associated mortality. Options include conservative, endoscopic, and surgical treatments. Conservative and endoscopic therapies, followed by surgical treatment are recommended to control the infection, to close the fistula, and to obliterate the empyema space.^[6,7]^ Conservative therapy is simple, safe, and noninvasive, including supportive treatments and some drainages. Adequate pleural drainage remains the cornerstone of empyema treatment. If BPF with empyema is diagnosed, it should be drained as soon as possible to prevent aspiration pneumonia and to control life-threatening sequela such as tension pneumothorax, aspiration, and respiratory failure. Different drainage methods have been described. Closed chest tube drainage was advocated as the first step in the treatment of Postpneumonectomy empyema (PPE), but the high failure rate demonstrates that chest tube drainage cannot control PPE effectively and increases the risk of contralateral lung inhalation and death.^[1]^ OWT has proven very useful, is credited with saving many lives, and is a simple technique that may be performed even in extremely unstable patients. Its effectiveness for providing adequate control of empyema, after total and partial lung resection, when combined with VAC therapy, has been demonstrated.^[3,4]^ The rate of recurrence has been reported to be 11% to 38.5% and an overall success rate of 84%.^[2]^ It can, however, lead to poor quality of life due to long-term dressing changes, compressive bandages, and thoracic pain, so we usually elect to perform debridement and rib resection drainage followed by antibiotic solution irrigation, which can achieve a satisfactory result.^[5,6]^ Endoscopic therapy should be performed to promote fistula closure after thoracic drainage because fistula closure is the key to a successful surgical outcome.^[6]^ It was including the bronchial mechanical abrasion, submucosal injection of absolute ethanol or polidocanol, endoscopic placement of biological glue, coils, and silver nitrate; the placement of covered stents, endobronchial valves, and an Amplatzer device. The degree of endoscopic success is variable and depends on the patient's underlying disease and the proximity and size of the fistulas. It has proven efficient for closing the fistula, is the treatment of choice for a small BPFs <6 mm in diameter (even in critically ill patients), but it is not suitable for patients with serious infection in the pleural cavity and larger fistulas. The success rate was 22.5% to 96.9%.^[7,8]^ Failure of endoscopic treatment does not preclude subsequent successful surgical management as it may be used as a bridge to surgical treatment, like our patients, or as a hybrid technique for a BPF >6 mm.^[7]^

Obliteration of BPF with empyema may be achieved by surgical treatments such as thoracoplasty and muscle flap transfer. Thoracoplasty has proved to be a reliable filling procedure, but its disadvantages reportedly include progressive scoliosis, chronic pain, progressive pulmonary insufficiency, and a mutilating cosmetic appearance, so the procedure has become obsolete.^[2]^ Pedicled muscle flaps were ideal for filling a contaminated space because of their good blood supply and their ability to reach almost any location in the pleural space. Depending on the anatomic location and size of the space, suitable muscle flaps could be mobilized for closing BPFs and filling the entire pleural space. The most common muscles used are the latissimus dorsi, serratus anterior, pectoralis major, pectoralis minor, and intercostal muscles. Among these, the latissimus dorsi muscle flap is the largest and most reliable.^[9,10]^ Latissimus dorsi muscle is a type V muscle according to its blood supply, thoracodorsal vessels, intercostals, or lumbar perforators. The proximal part of the muscle can be pedicled on the thoracodorsal vessels or the serratus branch. The thoracodorsal artery gives off 1 to 2 branches to the serratus muscle before entering the latissimus dorsi muscle. It can be elevated at full length and has a bulky tissue that provides reliable closure of a BPF, like in our patients.^[9,10]^ However, for patients who have previously undergone a posterolateral thoracotomy, the latissimus dorsi muscle or serratus anterior muscle flap may not be sufficient to obliterate the empyema cavity because they have already been divided during the original operation. In this situation, the pectoralis major and the pectoralis minor flaps are preferred.^[11]^ In patients with a large cavity, two or more muscle flaps can be transferred simultaneously for obliteration.^[12]^ In some cases, when pedicled muscle flap was not able to be used, a free musculocutaneous flap, such as a rectus abdominis flap, or a vastus lateralis flap, was harvested and/or the greater omentum was used, either alone or in combination.^[13–15]^

In conclusion, it is crucial to not only completely control infection and occlude bronchopleural fistulae, but also obliterate the empyema cavity. Thus, pedicled muscle flap transfer combined with conservative and endoscopic therapies for bronchopleural fistula with empyema, is a useful option for treatment in select patients. Our results show that it is a feasible and efficient surgical procedure with promising results.

## Author contributions

**Conceptualization:** Xiaowen He.

**Data curation:** Zhongliang He, Lifeng Shen, Weihua Xu.

**Formal analysis:** Zhongliang He.

**Investigation:** Zhongliang He.

**Resources:** Zhongliang He, Lifeng Shen.

**Supervision:** Lifeng Shen, Weihua Xu, Xiaowen He.

**Validation:** Xiaowen He.

**Visualization:** Weihua Xu, Xiaowen He.

**Writing – original draft:** Zhongliang He.

**Writing – review & editing:** Lifeng Shen, Weihua Xu, Xiaowen He.

## Abbreviations

Abbreviations: BPF = bronchopleural fistula, CT = computed tomography, MRI = magnetic resonance imaging, OWT = open window thoracostomy, POD = postoperative day, VAC = vacuum-assisted closure.
